# One-Year Effects of Omega-3 Treatment on Fatty Acids, Oxylipins, and Related Bioactive Lipids and Their Associations with Clinical Lipid and Inflammatory Biomarkers: Findings from a Substudy of the Vitamin D and Omega-3 Trial (VITAL)

**DOI:** 10.3390/metabo10110431

**Published:** 2020-10-27

**Authors:** Olga V. Demler, Yanyan Liu, Heike Luttmann-Gibson, Jeramie D. Watrous, Kim A. Lagerborg, Hesam Dashti, Franco Giulianini, Mallory Heath, Carlos A. Camargo, William S. Harris, Jay G. Wohlgemuth, Allen M. Andres, Saumya Tivari, Tao Long, Mahan Najhawan, Khoi Dao, James G. Prentice, Julia A. Larsen, Olivia I. Okereke, Karen H. Costenbader, Julie E. Buring, JoAnn E. Manson, Susan Cheng, Mohit Jain, Samia Mora

**Affiliations:** 1 Division of Preventive Medicine, Brigham and Women’s Hospital, Harvard Medical School, Boston, MA 02115, USA; yliu47@bwh.harvard.edu (Y.L.); hgibson@hsph.harvard.edu (H.L.-G.); hdashti@bwh.harvard.edu (H.D.); fgiulianini@rics.bwh.harvard.edu (F.G.); jburing@rics.bwh.harvard.edu (J.E.B.); jmanson@rics.bwh.harvard.edu (J.E.M.); smora@bwh.harvard.edu (S.M.); 2 Department of Epidemiology, Harvard T.H. Chan School of Public Health, Boston, MA 02115, USA; CCAMARGO@PARTNERS.ORG (C.A.C.J.); olivia.okereke@mgh.harvard.edu (O.I.O.); 3 Department of Pharmacology, University of California San Diego, La Jolla, CA 92037, USA; jeramie.watrous@gmail.com (J.D.W.); kimalehmann@gmail.com (K.A.L.); aandres@health.ucsd.edu (A.M.A.); satiwari1@gmail.com (S.T.); tlong@health.ucsd.edu (T.L.); mahannajhawan98@gmail.com (M.N.); kldao@ucsd.edu (K.D.); mjain@ucsd.edu (M.J.); 4 Division of Cardiovascular Medicine, Brigham and Women’s Hospital, Harvard Medical School, Boston, MA 02115, USA; mheath1@bwh.harvard.edu; 5 Department of Emergency Medicine, Massachusetts General Hospital, Boston, MA 02114, USA; 6 OmegaQuant Analytics, Sioux Falls, SD 57106, USA; Bill@omegaquant.com; 7 Quest Diagnostics, San Juan Capistrano, CA 92673, USA; Jay.G.Wohlgemuth@questdiagnostics.com (J.G.W.); James.G.Prentice@questdiagnostics.com (J.G.P.); Julia.A.Larsen@questdiagnostics.com (J.A.L.); 8 Department of Psychiatry, Massachusetts General Hospital, Boston, MA 02114, USA; 9 Division of Rheumatology, Inflammation and Immunity, Brigham and Women’s Hospital, Harvard Medical School, Boston, MA 02115, USA; kcostenbader@bwh.harvard.edu; 10 Smidt Heart Institute, Cedars-Sinai Medical Ctr, Los Angeles, CA 90048, USA; Susan.Cheng@cshs.org

**Keywords:** omega-3, bioactive lipids, oxylipins, free fatty acids, metabolomics, lipids, inflammatory biomarkers, clinical trial

## Abstract

Omega-3 (n-3) treatment may lower cardiovascular risk, yet its effects on the circulating lipidome and relation to cardiovascular risk biomarkers are unclear. We hypothesized that n-3 treatment is associated with favorable changes in downstream fatty acids (FAs), oxylipins, bioactive lipids, clinical lipid and inflammatory biomarkers. We examined these VITAL200, a nested substudy of 200 subjects balanced on demographics and treatment and randomly selected from the Vitamin D and Omega-3 Trial (VITAL). VITAL is a randomized double-blind trial of 840 mg/d eicosapentaenoic acid (EPA) + docosahexaenoic acid (DHA) vs. placebo among 25,871 individuals. Small polar bioactive lipid features, oxylipins and FAs from plasma and red blood cells were measured using three independent assaying techniques at baseline and one year. The Women’s Health Study (WHS) was used for replication with dietary n-3 intake. Randomized n-3 treatment led to changes in 143 FAs, oxylipins and bioactive lipids (False Discovery Rate (FDR) < 0.05 in VITAL200, validated (*p*-values < 0.05)) in WHS with increases in 95 including EPA, DHA, n-3 docosapentaenoic acid (DPA-n3), and decreases in 48 including DPA-n6, dihomo gamma linolenic (DGLA), adrenic and arachidonic acids. N-3 related changes in the bioactive lipidome were heterogeneously associated with changes in clinical lipid and inflammatory biomarkers. N-3 treatment significantly modulates the bioactive lipidome, which may contribute to its clinical benefits.

## 1. Introduction

Observational and experimental studies have shown improved cardiovascular disease (CVD) outcomes for diets rich with omega-3 (n-3), motivating interest in randomized trials testing n-3 treatment. Although earlier studies suggested that dietary fish and n-3 fatty acid (FA) intake were inversely associated with CVD, other studies have shown inconsistent results, with some (but not all) trials finding benefits for CVD risk reduction [1,2,3]. The recently completed Vitamin D and Omega-3 Trial (VITAL, NCT01169259) reported that 840 mg of n-3 fatty acids, including 460 mg of eicosapentaenoic acid (EPA) and 380 mg of docosahexaenoic acid (DHA) over a median intervention period of 5.3 years did not significantly reduce the primary endpoint of major cardiovascular disease (CVD), which included stroke, but significantly decreased the risk of major coronary heart disease (CHD) events by 17%. In particular, myocardial infarction (MI) was decreased by 28% [4,5]. In another recent trial of high-risk secondary prevention patients (Reduction of Cardiovascular Events with Icosapent Ethyl—Intervention (REDUCE-IT)), a high prescribed dose of a highly purified eicosapentaenoic acid ethyl ester (4 gm/d) reduced the risk of CVD events by 25% in statin-treated high-risk patients with established CVD or multiple risk factors [6].

A better understanding of the mechanisms of n-3 action could provide insight into these conflicting results and suggest better treatment targets. N-3 fatty acids are polyunsaturated fatty acids (PUFAs) that have pleiotropic physiologic actions, including effects on FAs, oxylipins or other bioactive lipids that are pivotal to a host of biological processes, such as lipid metabolism, vascular function, and systemic inflammation [7,8,9,10]. These n-3 derived lipids exert anti-inflammatory effects independently, as well as by inhibiting pro-inflammatory n-6 PUFA-derived bioactive lipids [7]. Such bioactive lipids include eicosanoids and oxylipins, which are derivative products of oxidation of PUFAs that play key roles in immune response, cell growth regulation, modulation of the regional flow of blood to tissues and of blood pressure, among other functions [11]. Recent work suggests that hundreds of oxylipin and related species may exist in human circulation, though many remain poorly characterized [12].

**Figure A1:**
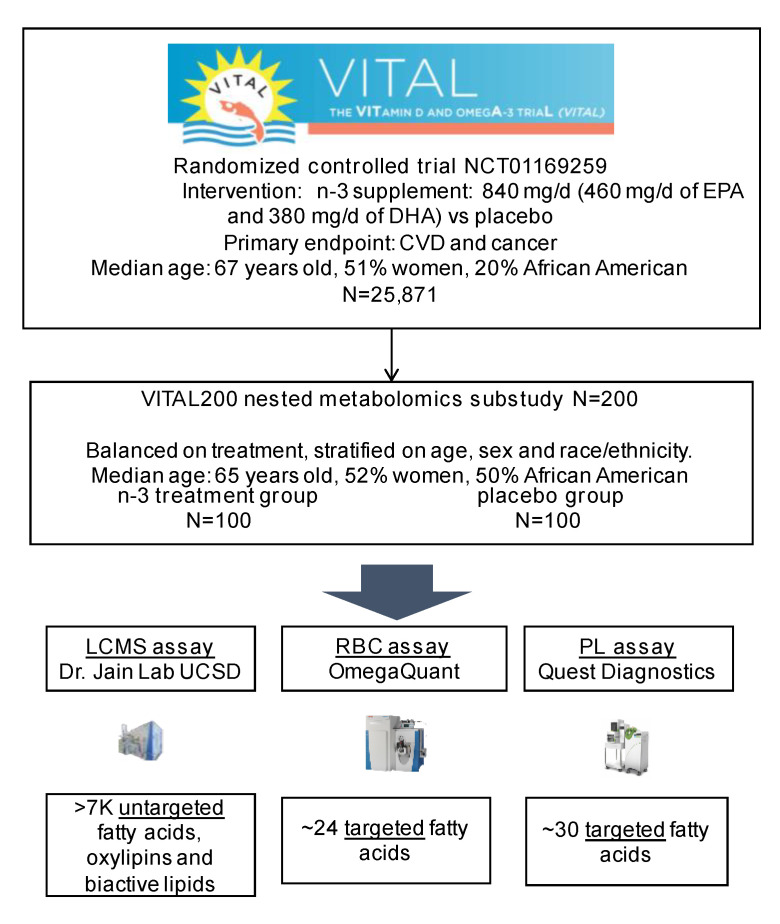
VITAL200 design. Assays: LCMS—high-throughput liquid chromatography–mass spectrometry of circulating free FAs, oxylipins and bioactive lipids from plasma; RBC—gas chromatography with flame ionization detection for red blood cells; PL—LCMS2 plasma phospholipids.

**Figure A2:**
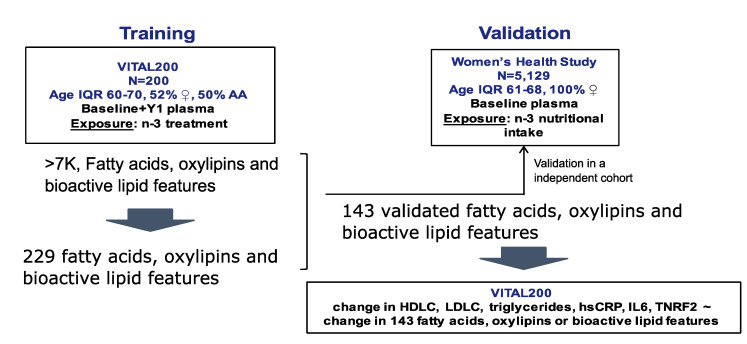
Discovery in the VITAL200 and validation in the Women’s Health Study (WHS).

We hypothesized that a randomized marine n-3 fatty acid treatment alters the balance of pro- and anti-inflammatory FAs, oxylipins or related bioactive lipids when compared to a placebo. To test this hypothesis, we used three different independent assaying techniques for measuring FAs and studied their baseline and one-year changes in response to n-3 randomized treatment in VITAL200. The three independent assaying techniques measured 24 FAs from red blood cells; 30 plasma phospholipids; and ~7000 circulating free fatty acids, oxylipins and small bioactive lipid features in plasma. In the Women’s Health Study (WHS), only the latter assaying technique was used in a subcohort of 5129 subjects. Therefore, we used WHS for validation using n-3 nutritional intake (Figure A1 and Figure A2) [13,14,15,16]. Validated FAs, oxylipins, and bioactive lipid features were then used to study associations of their one-year changes with changes in clinical lipid (low-density lipoprotein cholesterol (LDLC), high-density lipoprotein cholesterol (HDLC) and triglycerides) and inflammatory (high-sensitivity C-reactive protein (hsCRP), interleukin-6 (IL6) and tumor necrosis factor receptor 2 (TNRF2)) biomarkers in the VITAL200 study.

## 2. Results

### 2.1. Baseline Characteristics

Table 1 presents the demographic characteristics and clinical biomarkers of the VITAL200 (*n* = 200 subjects randomly selected from VITAL, balanced on age, sex and treatment assignment) and WHS participants (Appendix B.1 and Appendix B.2). In VITAL200 the median baseline age was 65 years old, with 52% women, about half African American, and 22% had prevalent diabetes. In WHS (*n* = 5129), participants’ median age was 64 years old, with 100% women, 4% diabetes, and predominantly white participants.

### 2.2. Baseline Levels of EPA, DHA and AA Were Consistent across the Three Assays

**Figure 1:**
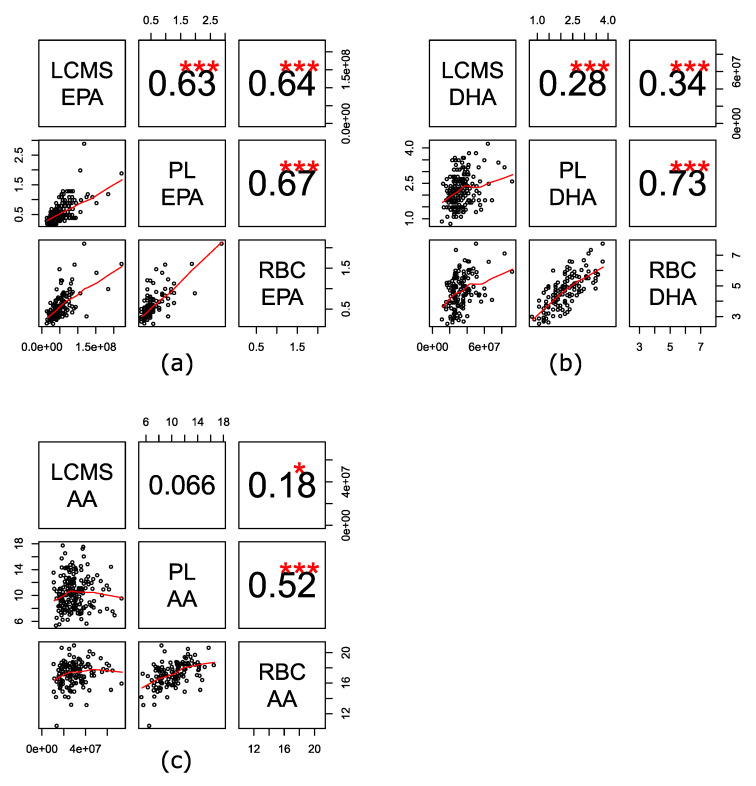
Spearman correlation of baseline levels of fatty acids (FAs). (**a**) Eicosapentaenoic acid (EPA); (**b**) docosahexaenoic acid (DHA); (**c**) arachidonic acid (AA) across three assays. Assays: LCMS—high-throughput liquid chromatography–mass spectrometry of circulating free FAs, oxylipins, and bioactive lipids in plasma; RBC—gas chromatography with flame ionization detection for red blood cells; PL—LCMS2 plasma phospholipids. *** 0 < *p*-value < 0.001, ** 0.001 ≤ *p*-value < 0.01, * 0.01 ≤ *p*-value < 0.05.

EPA, DHA and arachidonic acid (AA) in VITAL200 at baseline and at year one (about 400 samples total) were measured using three analytical assays of FAs from different lipid pools: high-throughput liquid chromatography–mass spectrometry non-targeted bioactive lipid platform (LCMS) was used to measure circulating free FAs, oxylipins, and bioactive lipids in plasma; gas chromatography with flame ionization detection for red blood cells (RBCs) was used to measure targeted FAs in RBC membranes; and a targeted LCMS2 assay was used to measure plasma phospholipid (PL) FAs. We observed a good correlation of measurements of free circulating EPA with EPA measured from circulating PL and RBCs (Spearman correlations 0.63–0.67, *p*-value < 0.001, Figure 1). Spearman correlations for DHA ranged between 0.28 and 0.73 across the three assays, with a good correlation for PL and RBC DHA (Spearman correlation 0.73, *p*-value <.001) and a moderate correlation with free DHA (Spearman correlation 0.28 and 0.34, *p*-value < 0.001). Correlations for free AA were weaker and ranged between 0.07 and 0.18 with PL and RBC AA, respectively, with a better correlation noted between PL and RBC AA (0.52, *p*-value < 0.001).

### 2.3. Baseline Levels of EPA, DHA and AA Were Consistent Across the Three Assays and in the n-3 Treatment and the Placebo Groups

**Figure 2:**
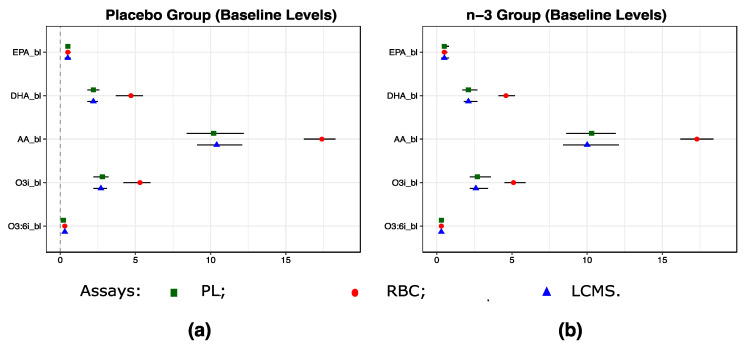
Baseline Levels of EPA, DHA, AA, o3i (EPA + DHA), and o3:6i (o3i:AA) according to the randomized treatment assignment in VITAL200. (**a**) in the placebo group; (**b**) in the n-3 treatment group. Because LCMS assay reports relative concentrations, baseline levels of EPA, DHA, AA, O3i, O3:6i were shifted and rescaled to match their medians and standard deviations observed for measurements from the plasma PL assay. Shapes and error bars denote medians and IQRs. n-3—omega 3 treatment group, EPA—eicosapentaenoic acid, DHA—docosahexaenoic acid, AA—arachidonic acid, O3i—omega3 index (RBC EPA + DHA expressed as a % of total RBC fatty acids), O3:6i—omega 3 to 6 ratio. Assays: LCMS—high-throughput liquid chromatography–mass spectrometry for circulating free FAs, oxylipins and bioactive lipids in plasma; RBC—gas chromatography with flame ionization detection for red blood cells; PL—LCMS2 plasma phospholipids.

Levels of EPA, DHA, AA, omega3 index (o3i = EPA + DHA), and omega3:6 index (o3:6i = [EPA + DHA]:AA) were similar when comparing baseline and year one measurements across three assays in placebo vs. the n-3 treatment groups because of randomized assignment to the two groups in the VITAL200 (Figure 2). Levels of these FAs measured using the three assays were comparable.

### 2.4. One Year of n-3 Treatment Lead to Changes of EPA, DHA and AA Levels in the n-3 Treatment vs the Placebo Group

**Figure 3:**
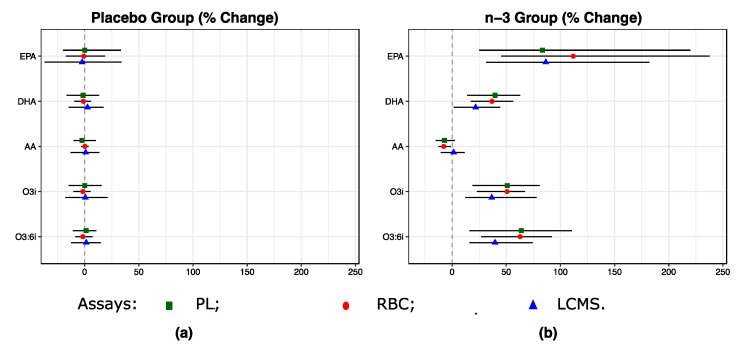
One-year % changes in EPA, DHA, AA, and combined levels according to the randomized treatment assignment (**a**) n-3 vs. (**b**) placebo in VITAL200. Shapes and error bars denote medians and IQRs. See footnote in Figure 2 and Abbreviations.

As expected, one year of n-3 treatment resulted in significant shifts in EPA, DHA and AA (Figure 3 and Table A1). EPA increased by 112%, 83% and 87% from baseline when measured by RBC, plasma PL and LCMS assays, respectively; DHA increased by 37%, 40% and 22%, respectively; and AA decreased by 8%, 7% when measured by RBC and PL assays and increased by 1% when measured by LCMS assay. Changes in composite measures such as o3i and o3:6i were also consistent across the three assays. For example, o3i and o3:6i increased by 50% to 64% on the RBC and plasma PL assays, respectively, and by 37% and 40% on the LCMS assay.

### 2.5. One Year of n-3 Treatment Lead to Significant Changes in 143 FAs, Oxylipins or Small Bioactive Lipid Features

**Figure 4:**
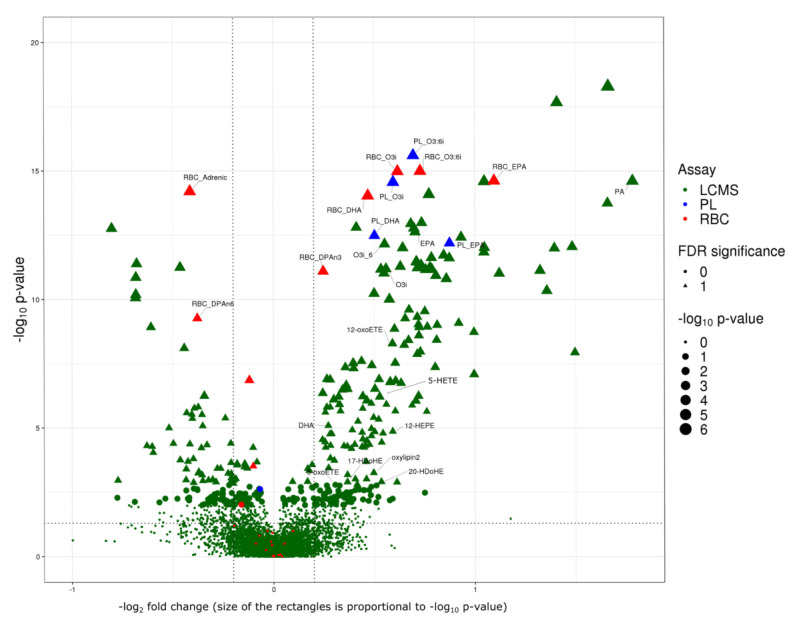
Volcano plot of fold changes from baseline to year one of FAs, oxylipins and small bioactive lipid features in response to randomized n-3 treatment in VITAL200. Seven bioactive lipid features with −log_2_(fold change) > 2.0 and −log_10_ (*p*-value) > 18 are not displayed. Fold changes were calculated as $median_{n-3\;treatment}\left(\frac{year1}{baseline}\right)/median_{placebo}\left(\frac{year1}{baseline}\right)$. Horizontal dashed line corresponds to *p*-value of 0.05, vertical dashed lines correspond to 20% change. False Discovery Rate (FDR) significance: significant controlling for False Discovery Rate at 0.05. *P*-values were estimated by Wilcoxon test. Assays: LCMS—high-throughput liquid chromatography–mass spectrometry of nontargeted circulating free FAs, oxylipins and bioactive lipids in plasma; RBC—gas chromatography with flame ionization detection for red blood cells; PL—LCMS2 plasma phospholipids.

In VITAL200, n-3 randomized treatment resulted in significant (FDR < 0.05) one-year changes in a total of 143 FAs, oxylipins and small bioactive lipid features. 134 of them were measured using the same LCMS assay in both cohorts: VITAL200 and WHS. Furthermore, nine additional FAs were measured using RBC and plasma PL assays in VITAL200 and showed effects that were similar to the LCMS assay (Figure 4). N-3 treatment resulted in significant one-year increase in 95 out of 143 FAs, oxylipins and small bioactive lipid features, five of them were annotated: EPA, DHA, o3i, o3:6i measured using all three assays and n-3 docosapentaenoic (DPA-n3) measured using the RBC assay (Figure 4) and an additional seven were putatively annotated oxylipins and FAs measured using LCMS assay: palmitic acid, 5-HEPE, 12-HEPE, 17HDoHE, 5-oxoETE and two novel putative oxylipins with unknown structure (standard not available). The median percent change in the n-3 treatment group ranged from 17% to 200% from baseline (Table A2). Of the 48 FAs, oxylipins and bioactive lipids and bioactive lipids whose levels decreased with n-3 treatment, four were annotated: AA, adrenic acid (docosatetraenoic cis ω6 polyunsaturated (22:4)), DPA-n6, and dihomo gamma linolenic (DGLA) (all measured using RBC assay) (Figure 3 and Table A2) with a percent change ranging from −29% to −5% from the baseline (Table A2).

### 2.6. Validation in the Women’s Health Study (WHS)

**Figure 5:**
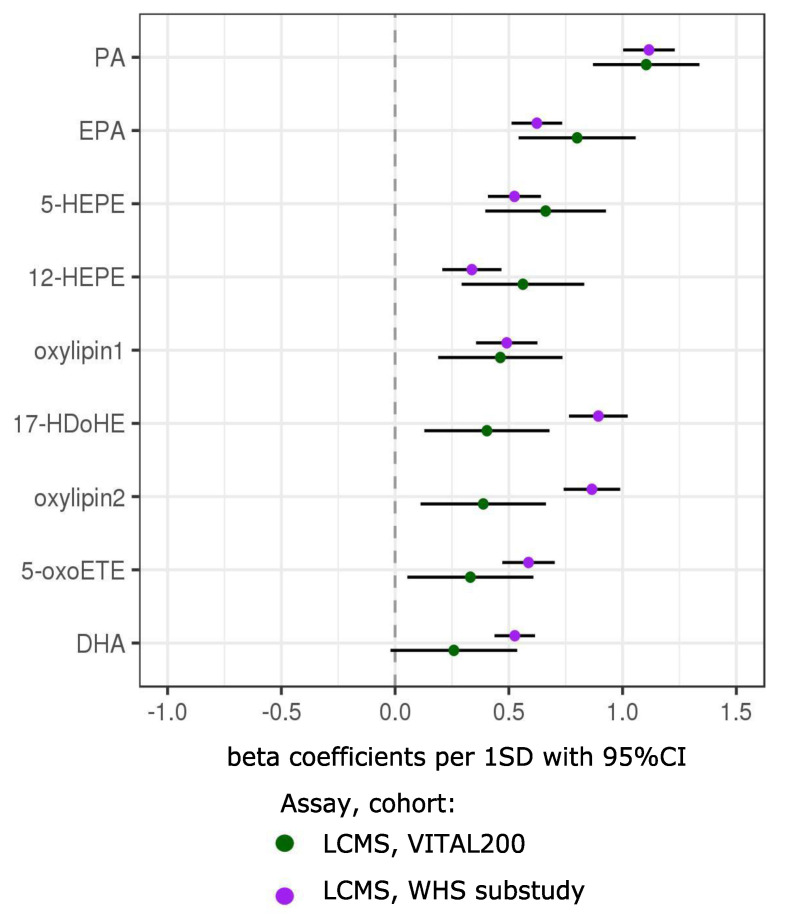
Validation in an independent cohort (the Women’s Health Study (WHS)) of significant (FDR < 0.05) associations between one-year changes in FAs, oxylipins and bioactive lipids with n-3 treatment in VITAL200. Associations between baseline levels with baseline nutritional n-3 intake in the WHS were used for validation. Only compounds with putative annotation using the LCMS assay are presented (LCMS assay—high-throughput liquid chromatography–mass spectrometry of circulating free FAs, oxylipins and bioactive lipids in plasma). Symbols with error bars are beta coefficients per 1SD with 95% confidence intervals. In VITAL200, longitudinal associations are modeled in one-way ANOVA with treatment group assignment as a predictor variable (sex and age were not significant and removed from the model). In the WHS, cross-sectional associations in linear regression were adjusted for age, sex and cardiovascular risk factors (high-density lipoprotein cholesterol (HDLC), total cholesterol, systolic blood pressure, treatment for hypertension, diabetes, current smoking). LCMS bioactive lipid features in VITAL200 were log transformed and their one-year changes were shifted to median 0 and standard deviation (SD) 1. In the WHS, batch effect-corrected baseline levels were log transformed and shifted and rescaled to median 0, SD1.

In order to validate above findings in an independent cohort, we examined associations of n-3-responsive FAs, oxylipins and bioactive lipids with respect to n-3 nutritional intake in an independent cohort of 5129 participants from the WHS. Of the significant small bioactive lipid features measured with the LCMS assay in VITAL200 (FDR < 0.05), 174 were also detected by the same LCMS assay in the WHS. Of these, 134 were successfully validated (*p*-value < 0.05 and had the same sign of association in the WHS with non-randomized n-3 nutritional intake) (Figure 5).

### 2.7. Associations of One-Year Changes in FAs, Oxylipins and Bioactive Lipids with One-year Changes in Six Downstream Clinical Biomarkers hsCRP, IL6, TNRF2, HDLC, LDLC, and Triglycerides

**Figure 6:**
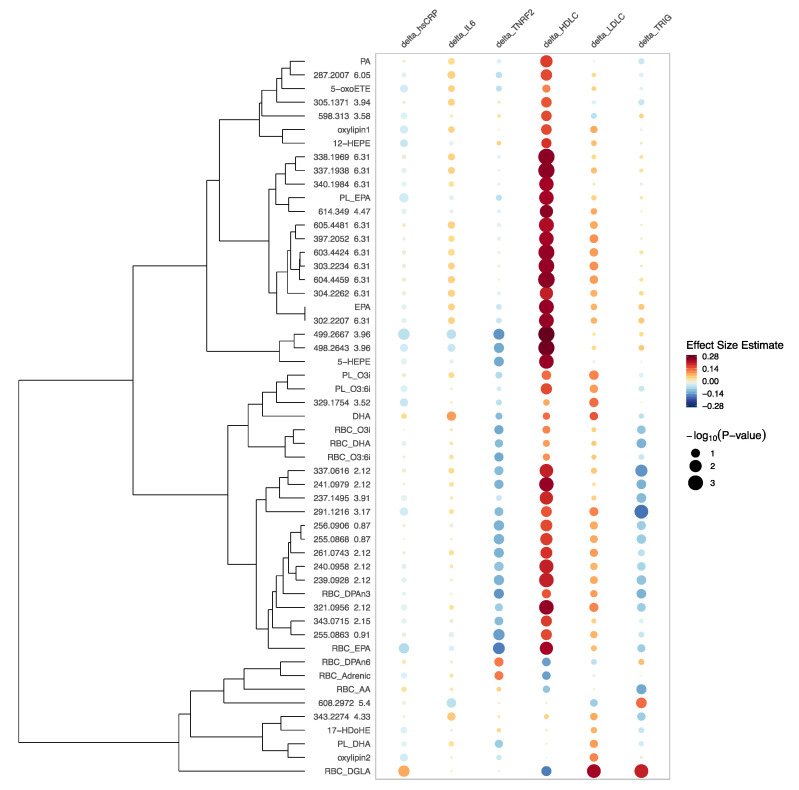
Rainplot [17] of beta coefficients of the significant associations of one-year changes in clinical biomarkers (high-sensitivity C-reactive protein (hsCRP), interleukin-6 (IL6), tumor necrosis factor receptor 2 (TNRF2), HDLC, low-density lipoprotein cholesterol (LDLC) and triglycerides) with one-year changes in FAs, oxylipins and bioactive lipid features controlling for age, sex and race. Only significant with n-3 treatment and annotated FAs, oxylipins and bioactive lipids are reported here as well as 30 out of 143 most significant un-annotated bioactive lipid features (labeled with *m*/*z* and RT) with respect to their association with n-3 treatment. “PL_” in front of the name means that compound was measured by plasma PL assay; “RBC_” in front of the name means that it was measured by RBC assay; otherwise the LCMS assay was used. Assays: LCMS—high-throughput liquid chromatography–mass spectrometry of circulating free FAs, oxylipins and bioactive lipids in plasma; RBC—gas chromatography with flame ionization detection for red blood cells; PL—LCMS2 plasma phospholipids. PA—palmitic acid, oxylipins 1,2 could not be annotated due to the lack of the standard in the library, they are novel oxylipins reported in [12].

Finally, we examined the changes in the n-3 responsive bioactive lipids in relation to concomitant changes in clinical lipid and inflammatory biomarkers measured from the same individuals and time points in VITAL200. As shown in Figure 6, we found variable associations between one-year changes in the FAs and bioactive lipids with one-year changes in six downstream clinical biomarkers of inflammation (hsCRP, IL6, TNRF2) and standard lipids (HDLC, LDLC, and triglycerides). Overall, we found that n-3 treatment led to changes in FAs, oxylipins and small bioactive lipid features that were associated more commonly with concomitant favorable increases in HDLC and decreases in triglycerides, and favorable decreases in the inflammatory biomarkers (most pronounced in TNRF2). By contrast, DPA-n6, DGLA, adrenic acid and AA (all measured by the RBC assay) formed a group for which one-year changes were inversely associated with changes in HDLC and positively associated with changes in TNRF2 (less so for DGLA), a pattern that is associated with increased cardiovascular risk. Changes in EPA and DHA were inversely associated with TNRF2 and positively associated with the HDLC, however these associations were somewhat attenuated for DHA. Changes in AA measured with the PL and the RBC assays and in adrenic acid measured with the RBC assay were positively associated with changes in all inflammatory biomarkers.

## 3. Discussion

In this nested substudy of a randomized clinical trial, we showed that randomized treatment with n-3 vs. placebo results in substantial changes in the bioactive lipidome. We identified 143 FAs, oxylipins or small bioactive lipid features that were significantly affected by one-year randomized treatment with n-3 vs. placebo. In total, 134 of those were measured using the LCMS assay, and their associations were successfully replicated in an independent prospective cohort (WHS) in relation to self-reported dietary intake of n-3; nine were measured using targeted assaying techniques (the plasma PL and the RBC). In total, 95 of the 143 FAs, oxylipins, or small bioactive lipid features were significantly increased with randomized n-3 treatment, while 48 FAs, oxylipins or small bioactive lipid features were decreased. All of the annotated FAs that decreased with n-3 treatment belong to the n-6 AA metabolic pathway. One-year changes in 143 FAs, oxylipins or small bioactive lipid features had heterogeneous associations with one-year changes in downstream clinical lipid and inflammatory biomarkers (HDLC, LDLC, triglycerides, hsCRP, IL6 and TNRF2).

We found that n-3 treatment generally leads to suppression of n-6 byproducts such as AA, DPA-n6, DGLA and adrenic acid by −25% to −6% (all measured by the RBC assay). Decrease in AA with EPA treatment was observed in The Japan EPA Lipid Intervention Study (JELIS) [18] and increase in EPA/AA ratio with n-3 treatment was observed in other studies [19]. The metabolisms of n-3 and n-6 share the same enzymes; therefore, higher intakes of the n-3 fatty acids may be suppressing the conversion of linoleic acid to its downstream metabolites which might explain the significant decrease in concentrations of n-6 by-products such as AA, DPA-n6, DGLA and adrenic acid. The level of AA is determined by the amount of AA metabolized by enzymes such as cyclooxygenase (COX) and the amount of AA extracted from phospholipids by phospholipase A2 (PLA2). It is possible that AA extracted from phospholipids by PLA2 does not compensate for the decrease in AA metabolized by COX, or it is possible that a different mechanism explains the reduction in AA in response to n-3 treatment [20,21].

EPA increased from baseline (by 112%, 83% and 87% using RBC, plasma PL and LCMS assays, respectively), which was a larger percent increase than that of DHA (37%, 40% and 22%, respectively). However, while the somewhat lower dose of DHA (375 mg vs. 465 mg for EPA, or 1:1.3 ratio) might have contributed to these differences in changes between EPA and DHA, it does not explain them fully, since the DHA change was disproportionately lower than the EPA change. Some of the metabolic by-products of n-3 FAs increased to a smaller degree than the EPA but to a similar degree as the percentage changes in the DHA: DPA-n3 (the RBC assay) increased by 17%, HDoHE by 30%, 12-HEPE by 43%, 5-HEPE by 50% (all measured with the LCMS assay). AA decreased by 8%, 7% (when measured by the RBC and the PL assays) and increased by 1% when measured by the LCMS assay, while some of the downstream n-6 FAs decreased to a larger extent such as DPA-n6 (−25%), adrenic acid (−24%) or to a comparable degree DGLA measured with RBC assay (−6%) (Appendix A Table A2).

The degree of correlation for EPA, DHA, AA varied across the different stores of FAs. As such, EPA, DHA, and AA in plasma phospholipids (the PL assay) correlated well with FAs in red blood cells (the RBC assay). However, when using LCMS assay only free EPA had a comparable strength of correlation with PL EPA and RBC EPA. Free DHA (the LCMS assay) had a weaker correlation with PL or RBC DHA, while free AA was uncorrelated with plasma phospholipid AA and only weakly correlated with RBC AA.

In general, we observed that one-year treatment with n-3 led to changes in FAs, oxylipins or small bioactive lipid features that were diversely associated with changes in inflammatory biomarkers (hsCRP, IL6 and TNRF2) with mostly negative associations with changes in TNRF2 and, to a smaller degree, with hsCRP. N-3 treatment boosts the generation of n-3 compounds that are important in resolving inflammation, cell signaling, and cardiovascular cell functioning and suppresses by-products of n-6 from the AA inflammatory cascade, an important part of the pro-inflammatory response [21]. Therefore, n-3 treatment promoted compounds from the n-3 pathway and suppressed compounds from the n-6 pathway, which might explain inverse associations for some of 143 FAs, oxylipins or small bioactive lipid features with inflammatory biomarkers. In contrast, changes in some of the measured n-6 by-products were positively associated with some of the inflammatory biomarkers. As such, changes in DGLA measured with the RBC assay were significantly associated with an increase in hsCRP (Figure 6). This observed heterogeneity of the association with inflammatory biomarkers might explain why in other VITAL study association of inflammatory biomarkers with n-3 treatment was significant in subgroups but not overall [22].

Effects in relation to clinical tests of cholesterol and triglycerides were even more variable. Of the 143 FAs, oxylipins, and bioactive lipid features measured by the LCMS assay that were modulated by randomized n-3 treatment in VITAL200, most were correlated with decrease in triglycerides and increases in HDLC and (to a smaller degree) LDLC. These results are consistent with prior knowledge about the relationship of n-3 FAs with lipid and lipoprotein levels. Triglyceride-lowering effects of n-3 treatment have been demonstrated in prior studies; however, the current findings suggest that there may be variable effects on triglycerides depending on the concomitant FA and related metabolite changes. Additionally, these results indicate that changes in some of the downstream FAs, oxylipins and bioactive lipids might have a stronger association with a decrease in triglycerides than EPA and DHA. These compounds should be investigated further as n-3 supplements are predominantly clinically used for their triglyceride-lowering effects.

Further research is needed to address whether n-3 therapy should be recommended for primary prevention of cardiovascular disease. In the VITAL, the primary cardiovascular endpoint (a composite endpoint of coronary heart disease, stroke, and cardiovascular mortality) was not significantly reduced with n-3 treatment (hazard ratio (HR) 0.92 95% confidence interval (CI) (0.80–1.06)), but n-3 treatment in the VITAL significantly reduced myocardial infarction (a prespecified secondary endpoint) by 28%, and significantly reduced total coronary heart disease events by 17%. These effects were even more pronounced in certain subgroups, especially among African-Americans and in particular for MI among African Americans, who have one cardiovascular risk factor (HR 0.28, 95% CI (0.08–0.99)) or 2+ risk factors (HR 0.16, 95% CI (0.05–0.45)) unadjusted for multiple comparisons [5]. Finally, these results in the VITAL are consistent with the results of the REDUCE-IT trial, which, unlike the VITAL, enrolled a higher risk secondary prevention cohort. A recent meta-analysis that included the results of both VITAL and REDUCE-IT showed a ~10% overall benefit for randomized n-3 treatment in lowering risk of coronary heart disease and myocardial infarction [1]. The current biomarker study sheds some light on that benefit, since we found that n-3 therapy leads to substantial changes in many fatty acids, oxylipins and bioactive lipids, which are also associated with changes in downstream clinical biomarkers that were mostly favorable for cardiovascular disease risk.

The strengths of this study include the use of two independent cohorts, control of confounding by experimental design that included a randomized trial, and the demonstration of concordance of results across three independent assays two of which (RBC and plasma PL) are targeted assays. Furthermore, this is the first study with one-year longitudinal measurements of clinical biomarkers and with metabolomic profiling of >7K FAs, oxylipins or small bioactive lipid features using high-throughput LCMS assay as well as RBC and plasma PL assays on the same samples. The results of this VITAL200 study, as a random sample of a large double-blind placebo-controlled trial, are less prone to confounding. The use of baseline and one-year measurements allowed us to minimize between-person heterogeneity. Additionally, we validated the VITAL LCMS results in an independent cohort (the WHS) with nutritional n-3 intake. Several oxylipins were assayed independently on all three assays and demonstrated consistency in the results across three assays.

The limitations of our study include possible confounding of the associations with the nutritional intake in the WHS; however, we used a prospective well-established cohort to minimize confounding and adjusted for potential confounders. EPA, DHA, and AA had high inter-assay correlations across the three assays, including targeted plasma PL and RBC assays, in the VITAL200 substudy, which confirms the validity of measurements using the untargeted LCMS assay. Many of the LCMS bioactive lipids are unknown or have putative annotations but all had consistent effects with n-3 nutritional intake in the WHS.

In summary, we found that randomized n-3 treatment in VITAL200 led to a cascade of changes in the bioactive lipidome. N-3 treatment led to significant one-year changes in the levels of 143 FAs, oxylipins or small bioactive lipid features. These findings were replicated in the WHS (an independent cohort) with n-3 nutritional intake. In addition, changes in these 143 FAs, oxylipins or small bioactive lipid features had heterogeneous associations with changes in downstream clinical lipid and inflammatory biomarkers. Most were inversely associated with changes in inflammatory biomarkers, especially with TNRF2 and hsCRP. Many were correlated with increases in lipid levels, in particular HDLC and LDLC, while several others (but not all) were associated with a concomitant decrease in triglycerides. Changes in several of the downstream FAs, oxylipins or bioactive lipids features might be associated with a stronger decrease in triglycerides than corresponding changes in EPA and DHA. These results indicate that n-3 treatment results in major changes in the bioactive lipidome, which, in turn, have mostly inverse associations with one-year changes in inflammatory biomarkers and heterogeneous associations with changes in standard lipid biomarkers including plasma triglycerides. A detailed profiling of n-3 and n-6 by-products is crucial for establishing a more refined picture of their metabolic effects on human health and should be examined in future studies.

## 4. Materials and Methods

### 4.1. Study Design

We assayed FAs, oxylipins, or small bioactive lipid features profiled with three independent assays using tandem baseline and one-year samples collected for the VITAL200 substudy, and validated results with one of the assays using the baseline samples of the WHS (*n* = 5129) (Figure A1 and Figure A2).

Participants for VITAL200 (*n* = 200) were selected randomly from participants who provided baseline and one-year blood samples from a VITAL ancillary study (Appendix B.1). Participants were stratified on age, sex, race/ethnicity (African American vs. white) and treatment arm. Briefly, VITAL (*n* = 25,871) is a recently completed randomized, double-blind, placebo-controlled trial (U.S. men aged ≥ 50 and women aged ≥ 55, including 5106 African Americans). The trial used factorial design to test effects of marine n-3 fatty acids (Omacor^®^, Pronova, Kalundborg, Sweden, and BASF, Ludwigshafen, Germany, 840 mg of 1.3:1 ratio of EPA:DHA) and/or vitamin D (2000 IU/d) supplements vs. placebo in the primary prevention of CVD and cancer [4,5]. Baseline (November 2011–March 2014) and follow-up questionnaires were administered to assess demographic, anthropometric, clinical risk factors, treatment compliance, and side effects. Sex, age, race/ethnicity, use of non-randomized supplements or medications, smoking, history of hypertension, and other relevant aspects of health history were collected from baseline questionnaires. Self-reported weight and height were recorded, and body-mass index (BMI) was calculated. At the time of enrollment, participants provided written informed consent.

The WHS substudy (median age at baseline 64 IQR (61–68)) consists of the *n* = 5129 participants who reached the age of 80 at follow-up and whose baseline plasma samples (1993–1996) were profiled using LCMS assay. The WHS was a randomized, double-blind, placebo-controlled trial (*n* = 39,876) designed to evaluate the balance of benefits and risks of low-dose aspirin and vitamin E in the primary prevention of cardiovascular disease and cancer in women aged 45 years or older and without a history of cardiovascular disease or cancer (other than nonmelanoma skin cancer), with no significant effects of these agents on the primary cardiovascular endpoint of the WHS [13,14,15,16].

The study protocol was approved by the Partners Institutional Review Board, Boston (protocol numbers 2017P001068, 2015P001069 and 2005P001414). 

### 4.2. Biomarker Profiling

VITAL200 baseline and one-year levels of FAs, oxylipins or small bioactive lipid features were assayed on three assays: (1) RBC: gas chromatography with flame ionization detection reporting levels of 24 FAs in RBC membranes, each reported as a percent of total FAs; (2) PL: liquid chromatography-mass spectrometry of about 30 plasma phospholipid FAs, each plasma phospholipids FA’s concentration is expressed as a percent of the total FAs present in each sample); (3) LCMS: high-throughput liquid chromatography-mass spectrometry of circulating free FAs, oxylipins and bioactive lipids in baseline and one-year plasma samples (reporting relative intensities of 7069 FAs, oxylipins or small bioactive lipid features). The latter LCMS assay was also used to assay the baseline plasma samples in the WHS. In VITAL200, baseline and one-year samples (in random order) were blindly assayed in tandem for all three assays. Details of these three methods, as well as the methods for measuring established clinical downstream lipid and inflammatory biomarkers (LDLC, HLDC, triglycerides, hsCRP, IL6 and TNRF2), are presented in Appendix B.4.

### 4.3. Statistical Analysis

FAs, oxylipins, and small bioactive lipid features were log-transformed, shifted, and rescaled to median 0 and standard deviation 1. Bioactive lipid features with more than 20% of missing values were removed from the analysis. Missing values of the remaining bioactive lipid features were imputed to 1/4 of the lowest observed value. Since baseline and one-year plasma samples were placed on neighboring wells, therefore batch effects and instrument drifts had minimal effect on one-year changes in bioactive lipid features by design. The benefits of this paired design were confirmed by high across assays correlations of the deltas (see Results) and the non-significance of the plate effect. Therefore, to avoid overcorrecting, we have not applied batch correction methods for the analysis involving changes from baseline in bioactive lipid features. In the analysis focused on baseline levels, logarithms of relative concentrations of all LCMS bioactive lipid features were corrected for plate effects, then shifted to a median 0 and SD 1. In plots and tables where LCMS bioactive lipid features (which have only relative quantification) were directly compared to bioactive lipid features measured with targeted assaying techniques (RBC and PL assays), we shifted and rescaled LCMS bioactive lipid features to match medians and standard deviations observed for their analogs assayed on the plasma PL assay.

#### 4.3.1. Association with n-3 Treatment

We used a discovery and validation approach to evaluate associations of FAs, oxylipins or small bioactive lipid features with n-3 treatment. In the discovery step, significance was established with Wilcoxon test. We adjusted for multiple comparisons by controlling for False Discovery Rate (FDR) at 5% using a resampling-based FDR method which accounts for correlation structure in the data [23,24,25]. Beta coefficients of the significant associations of each FAs, oxylipins or small bioactive lipid features with treatment in VITAL200 were estimated in univariate linear regression models with an n-3 treatment indicator as an independent predictor. As a sensitivity analysis, we re-ran univariate models used in the discovery analysis in VITAL200 while also adjusting for age, sex, race and plate. In the validation step, we tested the significant associations discovered above in the WHS, using baseline bioactive lipids measured with the same LCMS assay in relation to n-3 dietary intake. Linear regression models were adjusted for age, sex and additionally for CVD risk factors (HDL and total cholesterol, systolic blood pressure, treatment for hypertension, diabetes and current smoking).

#### 4.3.2. Associations of One-Year Randomized Changes in FAs, Oxylipins or Small Bioactive Lipid Features with One-year Changes in Downstream Clinical Lipid and Inflammatory Biomarkers

We also ran linear regression models using changes in one of the downstream biomarkers as an outcome and bioactive lipid as the predictor. One-year changes were log transformed, shifted to median 0 and an SD of 1, outliers above or below 3 SDs were removed, and models were adjusted for age, sex and race.

R version 3.5.3 was used to perform statistical analysis in this paper (R Foundation for Statistical Computing, Vienna, Austria. URL: https://www.R-project.org/) [26].

## Figures and Tables

**Table 1:** Baseline demographic characteristics and clinical biomarkers in the Vitamin D and Omega-3 Trial (VITAL) and the Women’s Health Study substudies.

VITAL200 Substudy				
	n-3 Treatment	Placebo	Total *	VITAL
N	100	100	200	25,871
Age, y	65 (60–71)	64 (60–69)	65 (60–70)	67 (63–71)
African American, n	50 (51%)	49 (50%)	99 (50%)	5106 (20%)
Women, n	53 (53%)	51 (51%)	104 (52%)	13,085 (51%)
BMI ^±^, kg/m^2^	29 (25–3 3)	28 (24–33)	28 (25–3 3)	27 (24–31)
HBPmed ^†^, n	63 (65%)	59 (62%)	122 (63%)	13166 (51%)
Diabetes, n	21 (21.2%)	23 (23%)	44 (22%)	3686 (14.3%)
Current Smoker, n	6 (6%)	7 (7%)	13 (7%)	1836 (7%)
Statin Treatment, n	35 (36%)	40 (40%)	75 (38%)	8890 (35%)
Total Chol, mg/dL	192 (169–210)	187 (168–220)	189 ^#^ (168–213)	204 (179–232)
LDLC, mg/dL	118 (100–1 36)	112 (96–143)	115 (96–138)	124 (102–148)
HDLC, mg/dL	47 (41–57)	48 (37–56.2)	47 (38–57)	52 (42–65)
Trig, mg/dL	108 (855–151)	110 (81–141)	109 (83–146)	111 (83–155)
hsCRP, mg/L	1.5 (0.8–4.0)	1.7 (0.8–4.1)	1.7 (0.8–4.1)	NA
IL6, pg/mL	1.7 (1.0–2.2)	1.7 (1.1–2.8)	1.7 (1.1–2.5)	NA
TNRF2, ng/mL	2651 (2104–3232)	2459 (2051–3201)	2530 (2068–3207)	NA
**Substudy of the Women’s Health Study (Validation Cohort)**				
	**Total**			
N	5129			
Age, y	64 (61–68)			
African American, n	78 (2%)			
Women, n	5129 (100%)			
BMI, kg/m^2^	25 (23–28)			
HBPmed ^†^, n	1093 (21%)			
Diabetes, n	199 (4%)			
Current Smoker, n	492 (10%)			
Statin Treatment, n	314 (6%)			
Total Chol, mg/dL	220 (195–245)			
LDLC, mg/dL	130 (109–154)			
HDLC, mg/dL	52 (43–63)			
Trig, mg/dL	133 (94–190)			
hsCRP, mg/L	2.4 (1.1–4.8)			
IL6, pg/mL	NA			
TNRF2, ng/mL	NA			

^#^ In VITAL lipid parameters were calculated in the sample of size 15,965; inflammatory biomarkers in a sample of 1561 subjects. N (%) or median (IQR); * None of the *p*-values of differences between groups were significant at 0.05 type 1 error (used the *t*-test for continuous variables, test of binomial proportion for count data); ^†^ high blood pressure (HBP) indicator was recorded in VITAL200 study and an indicator for high blood pressure treatment in the Women’s Health Study. ^±^ BMI—body mass index; LDLC—low density lipoprotein cholesterol; HDLC—high density lipoprotein cholesterol; hsCRP—high sensitivity C-reactive protein; IL6—interleukin-6; TNRF2—tumor necrosis factor receptor 2.

## Abbreviations

Abbreviation	Meaning
AA	arachidonic acid
adrenic acid	*cis* ω6 polyunsaturated (22:4) or 7,10,13,16-docosatetraenoic acid
CHD	coronary heart disease
CVD	cardiovascular disease
DGLA	dihomo gamma linolenic
DHA	docosahexaenoic acid
DPA-n6	docosapentaenoic n-6 acid
EPA	eicosapentaenoic acid
FA	fatty acid
FDR	False Discovery Rate
WHS	Women’s Health Study
GC	gas chromatography
RBC	gas chromatography-mass spectrometry with flame ionization decoupling in red blood cells
LC	liquid chromatography
LCMS	high-throughput liquid chromatography-mass spectrometry of circulating free FAs, oxylipins and bioactive lipids from plasma
plasma PL	LCMS2 plasma phospholipid assay
MI	myocardial infarction
MS	mass spectrometry
n-3	omega-3
n-6	omega-6
o3:6i	omega-3 to omega-6 index (=[EPA + DHA]/AA)
o3i	omega-3 index (=EPA + DHA)
SD	standard deviation
VITAL	Vitamin D and Omega-3 Trial

## Appendix A

**Table A1:** Significant one-year changes in fatty acid distribution according to the randomized treatment groups: n-3 vs. placebo.

	abs. Level at bl		abs. Change Y1−bl		% Change Y1−bl	
	Placebo	n−3 Treatment	Placebo	n−3 Treatment	Placebo	n−3 Treatment
**RBC Assay**						
N	84	75	74	63	74	63
EPA	0.5 (0.4:0.7) ^‡^	0.5 (0.4:0.7)	0 (−0.1:0.1)	0.7 (0.2:1.1)	−0.9 (−17.4:19) ^***^	111.8 (45.4:237.7) ^***^
DHA	4.7 (3.7:5.5)	4.6 (4.1:5.2)	−0.1 (−0.4:0.3)	1.7 (0.9:2.4)	−1.2 (−9.6:5.9) ^***^	36.8 (17.2:56.3) ^***^
AA	17.4 (16.2:18.3)	17.3 (16.2:18.4)	0 (−0.6:0.6)	−1.3 (−2.4:−0.2)	0.3 (−3.4:3.6) ^***^	−7.7 (−12.6:−1.4) ^***^
o3i	5.3 (4.2:6)	5.1 (4.5:5.9)	−0.1 (−0.5:0.3)	2.3 (1.2:3.2)	−1.7 (−10.4:5.5) ^***^	50.7 (22.9:67.2) ^***^
o3:6i	0.3 (0.2:0.4)	0.3 (0.3:0.4)	0 (−0.02−0.02)	0.2 (0.1:0.2)	−1.7 (−8.7:7.4) ^***^	62.8 (26.8:92.1) ^***^
**Plasma PL Assay**						
N	95	95	94	94	94	94
EPA	0.5 (0.4:0.6)	0.5 (0.4:0.8)	0 (−0.1:0.1)	0.5 (0.1:1)	0 (−20:33.3) ^***^	83.3 (25:220)
DHA	2.2 (1.8:2.6)	2.1 (1.7:2.7)	0 (−0.4:0.2)	0.8 (0.3:1.2)	−1.4 (−16.7:13.3) ^***^	39.6 (14:62.8) ^***^
AA	10.2 (8.4:12.2)	10.3 (8.6:11.9)	−0.2 (−1.1:1)	−0.7 (−1.6:0.2)	−2.5 (−10.1:10.2) ^**^	−7 (−15.1:2.7) ^**^
o3i	2.8 (2.2:3.2)	2.7 (2.2:3.6)	0 (−0.4:0.3)	1.3 (0.4:2.1)	0 (−14.8:15.6) ^***^	51 (18.8:80.9) ^***^
o3:6i	0.2 (0.2:0.3)	0.3 (0.2:0.3)	0 (−0.02−0.03)	0.2 (0:0.3)	1.3 (−10.9:10.7) ^***^	63.9 (15.9:110.7) ^***^
**LCMS Assay**						
N	100	99	100	98	100	98
EPA	0.5 (0.3:0.7)	0.5 (0.3:0.8)	0 (−0.2:0.2)	0.4 (0.2:0.7)	−2.3 (−36.9:34) ^***^	86.5 (31.5:182) ^***^
DHA	2.2 (1.8:2.5)	2.1 (1.8:2.7)	0 (−0.3:0.4)	0.5 (0:1)	2.6 (−14.8:17.5) ^***^	21.7 (1.4:44.4) ^***^
AA	10.4 (9.1:12.1)	10 (8.4:12.1)	0.1 (−1.4:1.2)	0.1 (−1.2:1)	0.9 (−12.9:13.5)	1.4 (−10.5:11.5)
o3i	2.7 (2.2:3.1)	2.6 (2.2:3.4)	0 (−0.5:0.5)	1 (0.3:1.7)	0.6 (−17.7:21.2) ^***^	36.6 (12:78.1) ^***^
o3:6i	0.3 (0.2:0.3)	0.3 (0.2:0.3)	0 (−0.04−0.04)	0.1 (0:0.2)	1.5 (−12.5:15) ^***^	39.6 (16.1:74.3) ^***^

Median (interquartile range (IQR)); ^‡^ Levels of FAs shown are % of total FAs. For example, in the placebo group, RBC EPA, constituted a median of 0.5% of all RBC FAs at baseline with IQR of (0.4%:0.7%). *** 0 < *p*-value < 0.001; ** 0.001 ≤ *p*-value < 0.01; * 0.01 ≤ *p*-value < 0.05; because LCMS bioactive lipid assay reports relative concentrations, baseline levels of EPA, DHA, AA, o3i, o3:6i measured on LCMS oxylipins assay were shifted and rescaled to match their medians and standard deviations observed for measurements from PL assay. Assays: LCMS—high-throughput liquid chromatography–mass spectrometry of circulating free FAs, oxylipins and bioactive lipids from plasma; RBC—gas chromatography with flame ionization detection for red blood cells; PL—LCMS2 plasma phospholipids.

**Table A2:** n-3 treatment effect on one-year percentage change in FAs, oxylipins and small bioactive lipid features in VITAL200.

Annotation or *m*/*z* RT	Assay	Median % Change	IQR
528.2974 5.38	LCMS	−29	(−52–20)
475.2657 4.13	LCMS	−26	(−47–18)
474.2627 4.13	LCMS	−26	(−47–19)
RBC_DPAn6	RBC	−25	(−39−10)
RBC_Adrenic	RBC	−24	(−32–7)
502.2943 5.62	LCMS	−24	(−44–5)
451.2623 4.42	LCMS	−20	(−47–6)
450.2628 4.43	LCMS	−20	(−47–7)
532.2654 4.43	LCMS	−19	(−46–9)
604.3627 5.07	LCMS	−17	(−40–2)
561.3002 5.73	LCMS	−13	(−32–5)
502.2874 4.59	LCMS	−12	(−24–5)
500.2787 4.62	LCMS	−12	(−25–5)
600.2039 4.8	LCMS	−11	(−22–5)
501.2818 4.61	LCMS	−11	(−25–4)
503.2859 4.8	LCMS	−10	(−28–2)
502.2846 4.8	LCMS	−10	(−26–0)
500.2794 4.8	LCMS	−10	(−24–0)
501.2821 4.8	LCMS	−9	(−23–1)
441.2109 2.3	LCMS	−9	(−25–5)
RBC_AA	RBC	−8	(−13−1)
583.2847 4.8	LCMS	−6	(−16–0)
RBC_DGLA	RBC	−6	(−11–2)
582.2814 4.8	LCMS	−5	(−16–2)
RBC_DPAn3	RBC	17	(5–34)
oxylipin1	LCMS	17	(−3–59)
527.286 4.69	LCMS	17	(−3–53)
326.2216 6.28	LCMS	20	(−10–82)
5-oxoETE	LCMS	23	(−11–66)
DHA	LCMS	24	(2–49)
364.2088 6.45	LCMS	28	(2–77)
363.209 6.44	LCMS	30	(1–78)
17-HDoHE	LCMS	30	(−6–113)
444.1595 2.2	LCMS	30	(−8–85)
365.2064 6.44	LCMS	33	(−1–87)
RBC_DHA	RBC	37	(17–56)
329.1724 4.91	LCMS	37	(−6–96)
325.218 6.28	LCMS	39	(−5–99)
PL_DHA	PL	40	(14–63)
12-HEPE	LCMS	43	(4–127)
oxylipin2	LCMS	45	(−5–130)
247.1704 5	LCMS	47	(−2–139)
5-HEPE	LCMS	50	(9–130)
247.1705 4.91	LCMS	50	(0–150)
RBC_O3i	RBC	51	(23–67)
PL_O3i	PL	51	(19–81)
EPA	LCMS	61	(24–131)
RBC_O3:6i	RBC	63	(27–92)
PL_O3:6i	PL	64	(16–111)
PL_EPA	PL	83	(25–220)
RBC_EPA	RBC	112	(45–238)
Palmitic Acid (Acetate Adduct)	LCMS	200	(23–456)

Percentage change was calculated as $100\%\left(oxylipin_{Y1}-\;oxylipin_{bl}\right)/oxylipin_{bl}$. IQR—interquartile range. Assays: LCMS—high-throughput liquid chromatography–mass spectrometry of circulating free FAs, oxylipins and bioactive lipids from plasma; RBC—gas chromatography with flame ionization detection for red blood cells; PL—LCMS2 plasma phospholipids. Annotations for LCMS assay are putative. FAs, oxylipins and small bioactive lipid features with annotations and top 30 un-annotated ones (based on *p*-values) are shown.

## Appendix B

### Appendix B.1. The Vitamin D and Omega-3 Trial (VITAL)

Vitamin D and Omega-3 Trial (*n* = 25,871) (VITAL, NCT01169259) is a completed large randomized, double-blind trial run between 2011 and 2017 evaluating marine n-3 fatty acid (Omacor^®^ 1 g per day) in the primary prevention of cardiovascular disease and cancer among US men (50+ years old) and women (55+ years old) [4,27]. Out of 25,871 randomized participants, 16,956 provided baseline blood samples and 1642 provided one-year blood samples as part of the ancillary study. As part of an ancillary VITAL biomarker study, a subset of participants who returned baseline blood during the winter or early spring of 2011–2012 and 2012–2013 were invited to additionally provide a follow-up blood sample 1 year later. In total, 200 subjects were selected out of these participants who provided baseline and one-year blood. Selection was performed by simple random sampling without replacement stratified by age, sex, race (African American or white), and treatment group assignment.

### Appendix B.2. The Women’s Health Study (WHS)

The Women’s Health Study (WHS), is a completed large randomized, double-blind, placebo-controlled trial (run between 1992 and 2004, observational follow-up still ongoing) originally evaluating low-dose aspirin, beta-carotene, and vitamin E in the primary prevention of CVD and cancer in women in a 2 × 2 × 2 factorial design [14,15,16]. The WHS participants were 39,876 apparently healthy female US health care professionals, ages 45 years or older, who were free of self-reported CVD (heart disease or stroke) and cancer at study entry. In total, 28,345 women provided baseline blood samples. A subsample of *n* = 5129 WHS participants were selected for high-throughput metabolomic profiling using nontargeted LCMS of circulating free FAs, oxylipins and bioactive lipids from plasma. The substudy included all individuals who had the chance to reach age 80 by the last follow-up: end of year 2014, and excluded those who were missing data on key demographic/clinical covariates, key biomarkers at baseline or who did not provide consent for blood to be stored for further biomarker analyses.

### Appendix B.3. Blood Sample Collection

Baseline blood sample collection in the VITAL200 followed the protocol of the main trial [4]. Briefly, a total of 16,642 (64.3% of randomized) VITAL participants provided a voluntary baseline pre-randomization blood sample. Participants were mailed a blood collection kit that contained a consent form, supplies and instructions for blood draw, and a cold pack and were returned via prepaid overnight courier. Upon arrival, samples were centrifuged and aliquoted into cryotubes as plasma, buffy coat, and red blood cells, and stored in liquid nitrogen freezers at a temperature of ≤ −130 °C. The process was completed within several hours of specimen receipt and the vast majority of samples were frozen within 30–36 h after venipuncture. As part of an ancillary biomarker study, a subset of participants (*n* = 1642) who returned baseline blood were invited to additionally provide a follow-up blood sample one year later. Baseline blood sample collection in the VITAL200 took place from November 2011 to April 2012, and one-year collection mainly from January 2012 to May 2013.

Pre-randomization blood samples from 28,345 women in WHS were frozen and stored for future analysis.

### Appendix B.4. Biomarker Profiling

#### Appendix B.4.1. Targeted Plasma Phospholipid Fatty Acids Measured with Plasma PL Assay

Quantifications of baseline and one-year levels 30 phosphorylated fatty acids including EPA, DHA and AA were measured with targeted plasma PL assay using the Thermo Fisher TSQ Quantum Ultra MS/MS System (Thermo Fisher Scientific, Waltham, MA, USA) by Quest laboratories (Nichols Institute, San Juan Capistrano, CA, USA). Each fatty acid concentration was determined based on peak area ratios (analyte vs. internal standards) using their respective standard curve. Fatty acid concentration is expressed as a percent of the total fatty acids present in each sample. CVs for the three measures ranged from 2.6% to 8.3% with an average CV of 5.4%.

#### Appendix B.4.2. Fatty Acids Measured with RBC Assay

RBC FAs composition was measured at Dr. William Harris’s lab at the South Dakota Technology Business Center, Sioux Falls, SD, USA (OmegaQuant)). The assay uses Gas Chromatography (GC) with flame ionization detection for RBCs [28]. Quantifications of baseline and one-year levels of 24 phospholipid membrane FAs from RBC were measured and included EPA, DHA and AA. No free FAs, and only trace amounts of FAs of cholesterol esters and triglycerides were included. First, fatty acids were converted to their corresponding fatty acid methyl esters to produce more volatile non-polar derivatives, acceptable for GC analysis. FAs composition was expressed as a weighted percent of total identified FAs. About 20% of the samples were excluded from the analysis because of the degradation due to storage conditions [29]. CVs were 3.29% (for EPA), 2.41% (for DHA), and 1.31% (for AA). The inter-run coefficient of variation (CV) for the major PUFAs (18:2w6, 20:4w6, 22:5w3, 22:6w3, and 20:5w3) is <4.9%; the intra-run CV is <5.4%.

#### Appendix B.4.3. Non-targeted Metabolomic Measurements with LCMS Assay

A panel of 7049 FAs, oxylipins and bioactive lipids were assayed following the same protocol at all stages of data collection in VITAL200 (*n* = 198 subjects, plasma samples at baseline and at one-year) and WHS (*n* = 5129, plasma samples at baseline) using LCMS Bioactive Lipids assay (Dr. Mohit Jain Lab, UCSD, San Diego, CA, USA) (LCMS assay). A directed non-targeted mass spectrometry approach was used to measure relative intensities of small polar bioactive lipid features including FAs, and oxylipins as described in [12]. In addition to experimental and pooled plasma samples, a library of chemical standards (mostly from Cayman Chemicals) was run. Chemical standards have a known fragmentation MS2 pattern in the libraries. Features from the experimental samples were then matched to those of the chemical standards library based on their *m*/*z*, retention times and fragmentation mass spectrum. Tandem plasma samples from each participant (baseline or one-year) were randomly placed on a plate, with baseline and one-year plasma samples placed in neighboring wells on a plate in order to minimize effects of instrument drifts. Internal standards were inserted in each well and three pooled plasma samples we placed at the beginning in the middle and at the end of each plate to monitor for any drift in data quality. Annotations of bioactive lipids in VITAL200 and WHS datasets were verified further by examining the raw spectral data. Additionally, as bioactive lipids with high correlations can be features of the same compound, raw spectral data were used to resolve these instances in order to avoid the same compound appearing more than once in the dataset.

#### Appendix B.4.4. Novel Oxylipins 1,2,3 Measured on LCMS Assay

Novel oxylipins 1–3 were reported in [12] where their chemical similarity to other eicosanoids/oxylipins was established. Commercial standards for them are not available yet. For further details, please see [12] where oxylipins 1–3 were referenced as 319_22__5_31.mgf, 333_21__2_77.mgf and 343_22__4_31.mgf.
